# Effects of Normothermic Conditioned Microwave Irradiation on Cultured Cells Using an Irradiation System with Semiconductor Oscillator and Thermo-regulatory Applicator

**DOI:** 10.1038/srep41244

**Published:** 2017-02-01

**Authors:** Mamiko Asano, Minoru Sakaguchi, Satoshi Tanaka, Keiichiro Kashimura, Tomohiko Mitani, Masaya Kawase, Hitoshi Matsumura, Takako Yamaguchi, Yoshikazu Fujita, Katsuyoshi Tabuse

**Affiliations:** 1 Faculty of Pharmaceutical Sciences, Osaka University of Pharmaceutical Sciences, 4-20-1 Nasahara, Takatsuki, Osaka 569-1094, Japan; 2 Faculty of Engineering, Chubu University, 1200 Matsumoto-cho, Kasugai, Aichi 487-8501, Japan; 3 Research Institute for Sustainable Humanosphere, Kyoto University, Gokasho, Uji, Kyoto, 611-0011, Japan; 4 Department of Bioscience, Nagahama Institute of Bio-Science and Technology, 1266 Tamura-cho, Nagahama, Shiga 526-0829, Japan

## Abstract

We investigated the effects of microwave irradiation under normothermic conditions on cultured cells. For this study, we developed an irradiation system constituted with semiconductor microwave oscillator (2.45 GHz) and thermos-regulatory applicator, which could irradiate microwaves at varied output powers to maintain the temperature of cultured cells at 37 °C. Seven out of eight types of cultured cells were killed by microwave irradiation, where four were not affected by thermal treatment at 42.5 °C. Since the dielectric properties such as ε’, ε” and tanδ showed similar values at 2.45 GHz among cell types and media, the degree of microwave energy absorbed by cells might be almost the same among cell types. Thus, the vulnerability of cells to microwave irradiation might be different among cell types. In HL-60 cells, which were the most sensitive to microwave irradiation, the viability decreased as irradiation time and irradiation output increased; accordingly, the decrease in viability was correlated to an increase in total joule. However, when a high or low amount of joules per minute was supplied, the correlation between cellular viability and total joules became relatively weak. It is hypothesized that kinds of cancer cells are efficiently killed by respective specific output of microwave under normothermic cellular conditions.

Microwaves are a form of electromagnetic wave that can efficiently generate heat in target substances. Microwaves have been utilized extensively in many applications in industrialized society. In cancer therapies, efficient microwave heat generation has been applied in microwave coagulation therapy (MCT) and hyperthermia treatment. MCT is a surgical method by which tumors are ablated through microwave-mediated coagulation of cells, leading to cellular death in the treatment area and a subsequent reduction in tumor size^1^,^2^. Hyperthermia treatment is a thermal therapy in which the cancer region is heated via microwave irradiation at over 42.5 °C, resulting in cancer cell death^3^,^4^,^5^. Thus, these therapies kill cancer cells through high temperature and use microwaves only as a tool for heat generation.

Recent studies have shown that several chemical reactions are promoted by microwave irradiation at lower temperatures than those observed with conventional heating methods such as using an oil bath^6^,^7^,^8^. Additionally, biological phenomena are controlled by microwave irradiation whose conditions hardly generate heat^9^,^10^,^11^,^12^,^13^,^14^,^15^,^16^,^17^,^18^,^19^. A cancer therapy called “oncothermia” was developed recently in which cancer cells were killed under normothermic radio-wave irradiation conditions^20^,^21^,^22^. These phenomena cannot be simply attributed to the effects of high temperature, implying the existence of ‘non-thermal effects’ that can be derived from microwave irradiation.

Based on these reports, we hypothesized that cancer cells would be killed by microwaves at a lower temperature (37 °C) than that used for current cancer therapies. If cancer cells can be killed by microwave irradiation under normothermic conditions, this phenomenon could be applied to future cancer therapies. In doing so, the applicable range of the therapy would be expanded, and heat-related side effects would be avoided.

In biological research, various types of cultured cells have been investigated to determine whether or not physiological changes related to induction of cell death^9^,^11^,^16^,^17^,^18^, the cell cycle^9^,^10^,^11^, and gene expression^12^,^15^,^19^ occur upon exposure to microwave irradiation under normothermic conditions. However, because the purpose of these studies was generally to investigate the dangers of microwave irradiation from telecommunications devices, the range of the microwave irradiation was limited to that used in telecommunication devices.

In contrast, for microwave cancer therapies, magnetrons have been widely used as microwave oscillators. In clinical studies, morphological changes of hepatocellular tumors have been observed after MCT^23^,^24^. However, magnetrons produce a huge output^25^,^26^, and it is almost impossible to use them for microwave irradiation under normothermic conditions.

For the present study, we developed a novel microwave irradiation system that can provide microwave irradiation under normothermic conditions. This system consists of a semiconductor microwave oscillator and an applicator; thus, it can control the irradiation output and temperature of cultured cells precisely. Using this system, we examined the viability of cultured cells under microwave irradiation with normothermic conditions. Additionally, we investigated the relationship between the microwave energy absorbed into cells and cellular viability.

## Results

### Viability and Dielectric Properties of Cultured Cells under Microwave Irradiation

**Figure 1:**
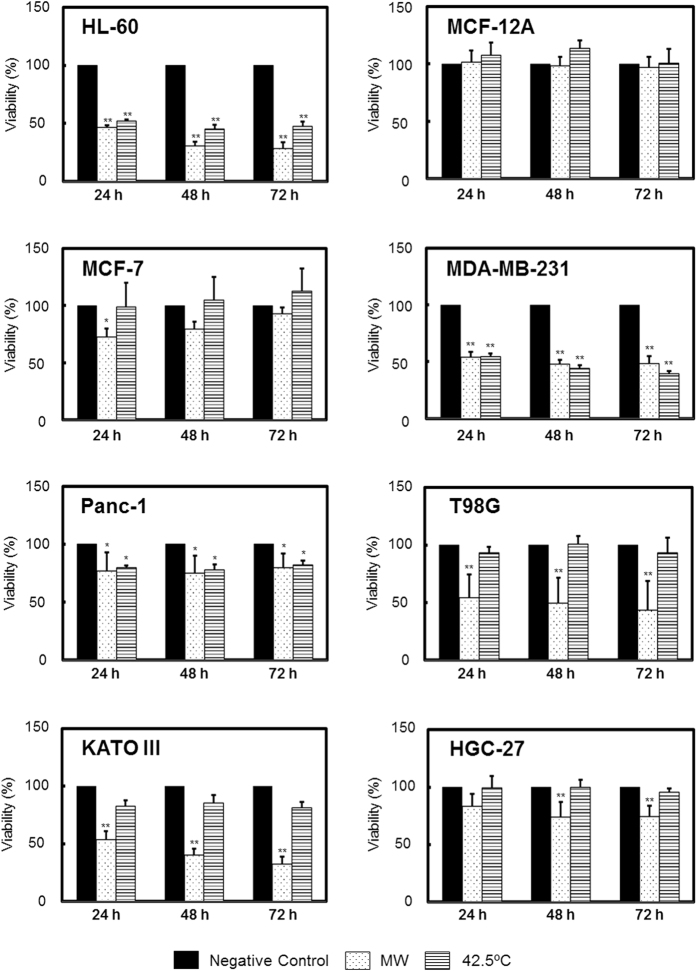
Viability of cultured cells under microwave irradiation. Microwave irradiation (described as ‘MW’) was applied for 1 h with the temperature of the cultured cells maintained at 37 °C, whereas the temperature inside the applicator was set at 10 °C. During irradiation, output could be generated at the maximum value of 20 W. After the irradiation ceased, cells were moved to a CO_2_ incubator and incubated for 24, 48, or 72 h. The negative control cells were incubated at 37 °C in a CO_2_ incubator rather than being subjected to microwave irradiation. The cells treated at 42.5 °C were incubated at an applicator temperature of 42.5 °C without receiving microwave irradiation. Cellular viability was determined using the WST-8 assay. Rates are shown relative to the absorbance of negative control cells, whose values were defined as “100”. The horizontal axes indicate the duration of incubation after irradiation. Data are expressed as the mean ± SD of four independent experiments. Asterisks indicate significant differences from the negative control: *P < 0.05, **P < 0.01.

We evaluated the viability of cultured cells under microwave irradiation in our irradiation system (Fig. 1). Microwave irradiation was applied for 1 h with the irradiation temperature maintained at 37 °C and the temperature inside the applicator set at 10 °C. After irradiation, cells were incubated in a CO_2_ incubator for 24, 48, and 72 h. As the thermal treatment, cells were incubated at 42.5 °C, whose temperature is well-known to be able to kill cells^27^. The viability of each cancer cell line except for MCF-12A was decreased significantly by microwave irradiation. In MCF-7, T98G, KATO III, and HGC-27 cells, viability was decreased by microwave irradiation even though the viability of cells incubated at 42.5 °C did not decrease significantly. In HL-60, MDA-MB-231 and Panc-1 cells, viability was decreased by both microwave irradiation and thermal treatment at 42.5 °C. The viability decreased the most in HL-60 cells, to 46.3% (24 h), 30.4% (48 h), and 28.3% (72 h), under microwave irradiation. The viability of MCF-12A cells was not affected by microwave irradiation or incubation at 42.5 °C.

**Figure 2:**
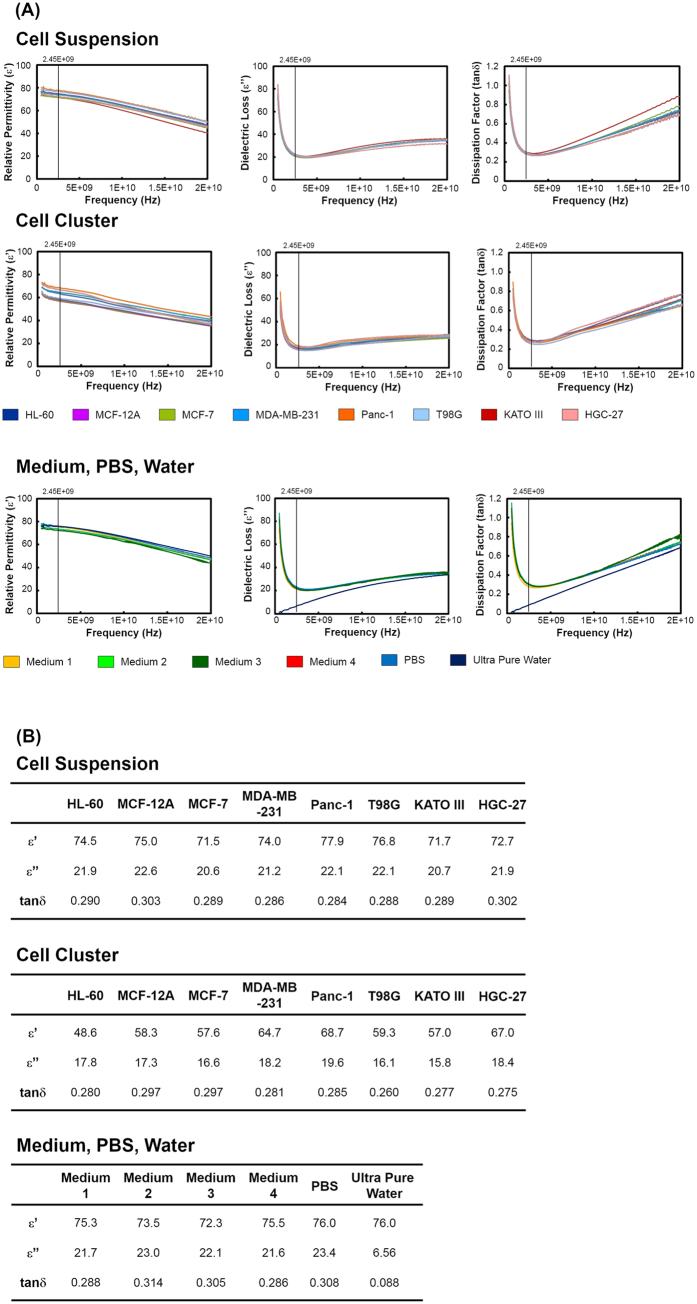
Dielectric properties of cultured cells. (**A**) Relative permittivity (ε’), dielectric loss (ε”), and dissipation factor (tanδ) at frequencies between 500 MHz and 20 GHz are shown for cell suspensions and cell clusters of the cultured cells, for the medium of the cultured cells, and for PBS and ultra0pure water. Medium 1 was for HL-60 cells, Medium 2 was for MCF-12A cells, Medium 3 was for HGC-27 cells, and Medium 4 was for other cultured cells. (**B**) Values of relative permittivity (ε’), dielectric loss (ε”), and dissipation factor (tanδ) at 2.45 GHz.

We also measured dielectric properties such as the relative permittivity (ε′) and dielectric loss (ε″) of the cultured cells at 500 MHz to 20 GHz, and calculated the dissipation factor (tanδ) (Fig. 2). The concentration of the cell suspension was the same as that in the experiment of Fig. 1. Phosphate buffered saline (PBS) and ultra-pure water were also measured for comparison. The maximum ε′ values for all samples were obtained at 500 MHz, and the values decreased as the frequency increased (Fig. 2A). The maximum values of ε″ and tanδ for all of the samples, except for ultra-pure water, were also obtained at 500 MHz, and they decreased as the frequency increased, but then increased again after the minimum value was obtained at approximately 2.45 GHz. The values of ε′, ε″ and tanδ at 2.45 GHz were not remarkably different, except for ultra-pure water (Fig. 2B).

### Viability of HL-60 cells Under Various Microwave Irradiation Conditions

**Figure 3:**
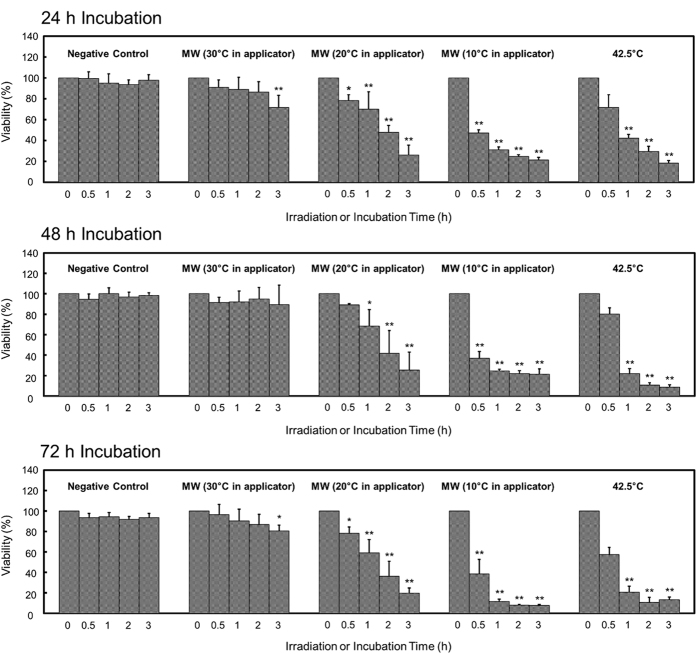
Viability of HL-60 cells under various microwave irradiation conditions. Microwave irradiation (described as ‘MW’) was applied for 0, 0.5, 1, 2, and 3 h with the temperature of the cultured cells maintained at 37 °C, whereas the temperature inside the applicator was set at 10, 20, or 30 °C. During irradiation, output could be generated at the maximum value of 20 W. After the cessation of irradiation, cells were moved to a CO_2_ incubator and incubated for 24, 48, or 72 h. The negative control cells were incubated at 37 °C in a CO_2_ incubator rather than being subjected to microwave irradiation. The cells treated at 42.5 °C were incubated in the applicator at 42.5 °C without being subjected to microwave irradiation. Cellular viability was determined using the WST-8 assay. Rates are shown relative to the initial absorbance, whose values were defined as “100”. The horizontal axes indicate the duration of irradiation. Data are expressed as the mean ± SD of four independent experiments. Asterisks indicate significant differences from 0 h: *P < 0.05, **P < 0.01.

HL-60 cells, which were the most sensitive to microwave irradiation in the experiments shown in Fig. 1, were subject to microwave irradiation under various irradiation conditions, and their viability was evaluated. Microwave irradiation was applied for 0, 0.5, 1, 2, and 3 h with the dish temperature maintained at 37 °C but the temperature inside the applicator set to 10, 20 or 30 °C. After irradiation, cells were incubated in a CO_2_ incubator for 24, 48, and 72 h. As shown in Fig. 3, the viability of cells subjected to microwave irradiation decreased as the irradiation time and output increased. At a temperature of 30 °C inside the applicator, the viability was not significantly decreased compared to the initial cell viability even with prolonged irradiation, except for 3 h irradiation and incubation for 24 and 72 h. In contrast, the viability at the temperatures of 10 and 20 °C inside the applicator was significantly decreased, except when using a 20 °C applicator temperature with 0.5 h irradiation and incubation for 48 h. In addition, the viability of cells incubated at 42.5 °C was also decreased, except for with the 0.5 h incubation.

### Correlation between Viability and Joule Heat in HL-60 Cells

**Figure 4:**
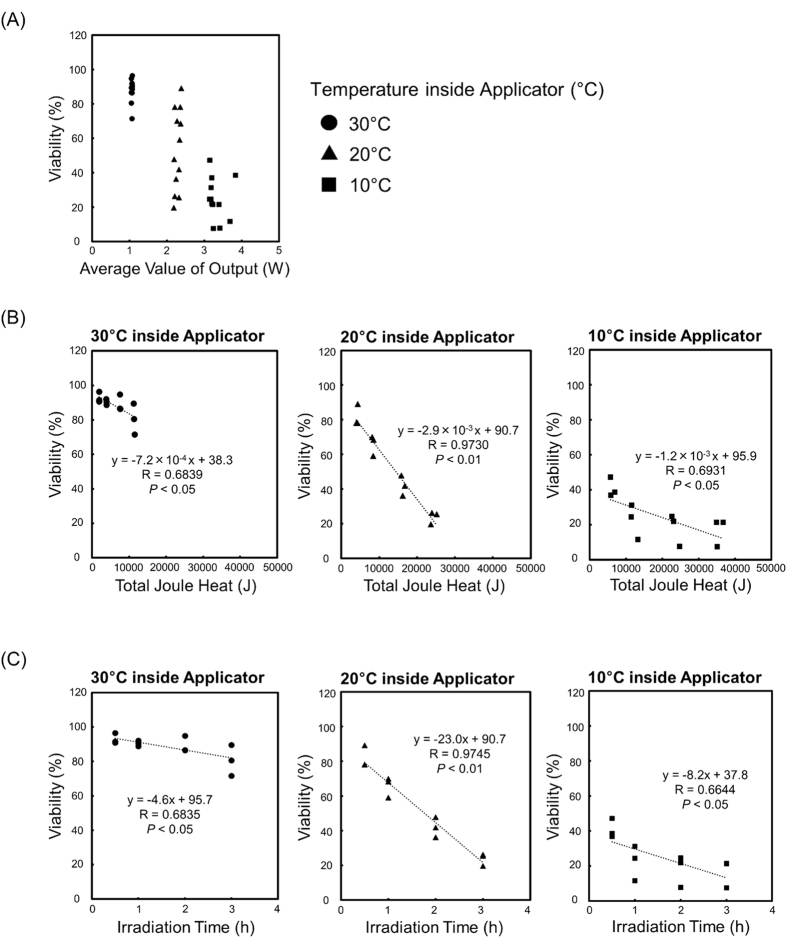
Correlation between viability and joule heat in HL-60 cells. (**A**) Correlation between viability of HL-60 cells and joule heat per minute, derived from the calculation for Fig. 3. (**B**) Correlation between viability of HL-60 cells and total joule heat for each temperature inside the applicator.

We calculated the joule heat for the experiments described in Fig. 3, and the relationship between the output and the cellular viability for each microwave condition (at applicator temperatures of 10, 20, and 30 °C) is shown in Fig. 4A. The mean values for the output were 3.3 W at 10 °C inside the applicator, 2.3 W at 20 °C inside the applicator, and 1.0 W at 30 °C inside the applicator. We also showed that the relationship between the total joule heat and the cellular viability for each microwave condition (at applicator temperatures of 10, 20, and 30 °C) in Fig. 4B. The total joule was calculated using the equation: W[J] = P[W] × t[s], where W, P, and t are the total joule [J], output energy monitored by our system [W], and irradiation time [s], respectively. At a temperature of 30 °C inside the applicator, the viability hardly decreased, even at prolonged a total joule heat. However, the viability at a temperature of 10 °C inside the applicator decreased dramatically even with only a small total joule. In contrast, the viability at a temperature of 20 °C inside the applicator was more strongly correlated to total joule heat than the viability at temperatures of 10 and 30 °C inside the applicator, and the linear regression equation was y = −2.9 × 10^−3^ x + 90.7 (correlation coefficient; R = 0.9730, *p* < 0.01).

## Discussion

The aim of this study was to investigate the effects of normothermic microwave irradiation on cultured cells. First, cultured cells were irradiated by microwaves under normothermic conditions that kept the temperature of the cultured cells at 37 °C, and the cellular viability was examined. The viability of cells other than MCF-12A cells was decreased by microwave irradiation, and the magnitude of the viability-decreasing effect was different among the types of cells. Specifically, the viability of MCF-7, T98G, KATO III, and HGC-27 cells was decreased by microwave irradiation but not by heating at 42.5 °C. These results indicated that the thermal-resistant cells could be killed by microwave irradiation under normothermic conditions.

In addition, even though the cells were derived from identical tissues, their responses to microwave and thermal stimuli were different. In breast cells, the viability of normal MCF-12A cells was not affected, whereas cancer cells, i.e., MCF-7 and MDA-MB-231 cells, were killed by microwave irradiation. In gastric cells, the viability of KATO III cancer cells was decreased drastically in contrast to that of non-cancer HGC-27 cells.

We hypothesized that this difference in cellular viability among the cell types might result from differences in the ability of cells to absorb microwave energy. Therefore, we measured the dielectric properties of cultured cells. With respect to radio wave irradiation, a relationship between dielectric properties and viability has been reported in some cell types, where cell death was reported to be significantly induced in proportion to the value of relative permittivity (ε’) and dielectric loss (ε”)^28^. Dielectric properties such as ε’, ε”, and tanδ did not vary remarkably among cell types or media. We therefore inferred that the degree of microwave energy absorbed by cells might be almost the same among cell types. Thus, the vulnerability of cells to microwave irradiation might be different among cell types. Of note, the value for ultra-pure water was different from that of the other samples, probably because of a lack of ionic conduction.

We examined the viability of HL-60 cells under various irradiation conditions to investigate the relationship between the amount of microwave energy applied and cellular viability. The viability of HL-60 cells decreased as irradiation time and irradiation output increased. Additionally, we calculated the Joule heat for the experiments of Fig. 3 to investigate the results in greater detail. The viability of cells decreased in correlation with the increase in total joule. However, the viability was decreased significantly under conditions of high microwave output and short irradiation time when an equivalent joule heat was supplied. These results suggest that the amount of output was a crucial factor and there is a threshold microwave energy required to induce cell death, especially considering that it is well known that ionizing radiation has a threshold energy level for inducing cell death^29^,^30^,^31^.

All experiments in this study used by a novel microwave irradiation system that we originally developed. The important characteristics of this system are that microwave irradiation can be applied at various irradiation outputs with precise control under normothermic conditions, which is difficult to achieve when employing typically used magnetron and telecommunication devices.

In conclusion, we showed that cell death was induced by microwave irradiation under normothermic conditions in various cultured cells. We also showed the relationship between the microwave energy and cellular viability. Our results would suggest the possibility of a future cancer therapy that kills tumors by microwave irradiation under normothermic conditions. In such a therapy, the side effects derived from heating would be avoided. Such a therapy might also be effective against tumors that cannot be killed by thermal treatment. Furthermore, breast tumors might be killed selectively without killing normal cells. Further studies are required to develop such a therapy.

## Methods

### Cell Growth and Conditions

The promyelomonocytic leukemia cell line HL-60 was donated by the National Hospital Organization, Osaka Minami Medical Center (Osaka, Japan). The human breast cancer cell line MDA-MB-231 (92020424) was obtained from the European Collection of Authenticated Cell Cultures (ECACC, Salisbury, UK). The human normal breast cell line MCF-12A (ATCC CRL-10782) was obtained from the American Type Culture Collection (ATCC, Rockville, MD). The human gastric cancer cell line KATO III (JCRB0611) was obtained from the Health Science Research Resources Bank (HSRRB, Osaka, Japan). The human breast cancer cell line MCF-7 (RCB1904), the human glioblastoma multiforme cell line T98G (RCB1954), the human pancreatic cancer cell line Panc-1 (RCB2095), and the mucin-producing human gastric cancer cell line HGC-27 (RCB0500) were provided by the RIKEN BRC through the National Bio-Resource Project of MEXT (Ibaraki, Japan).

HL-60 cells were grown in RPMI 1640 (Sigma-Aldrich, MO, USA) supplemented with 10% fetal bovine serum (FBS) and GlutaMAX (Thermo Fisher Scientific, Yokohama, Japan). MCF-12A cells were grown in Dulbecco’s modified Eagle medium/F-12 Ham medium (Nacalai Tesque, Kyoto, Japan) supplemented with 20 ng/ml human epidermal growth factor (Sigma-Aldrich, St Louis, USA), 0.01 mg/ml bovine insulin (Sigma-Aldrich, St Louis, USA), 500 ng/ml hydrocortisone (Nacalai Tesque, Kyoto, Japan), and 5% horse serum (Thermo Fisher Scientific, Yokohama, Japan). HGC-27 cells were grown in Minimum Essential Medium (MEM, Thermo Fisher Scientific, Yokohama, Japan) supplemented with 10% FBS. The other cell lines were grown in RPMI 1640 supplemented with 10% FBS. Cells were incubated at 37 °C in a humidified atmosphere with 5% CO_2_.

### Development of a Novel Microwave Irradiation System

**Figure 5:**
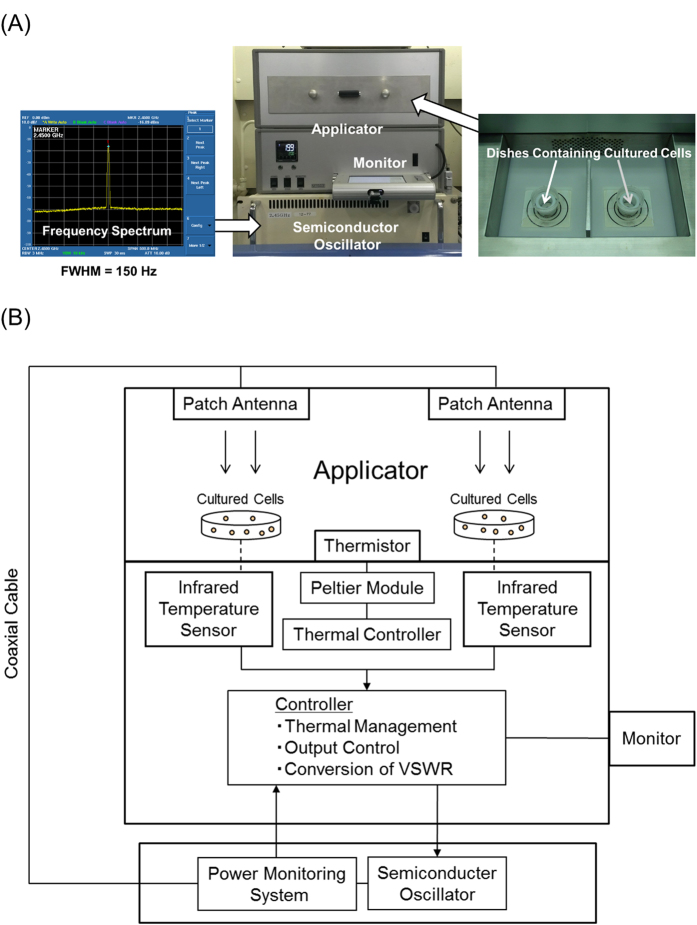
Novel irradiation system. (**A**) Photographic appearance: the novel irradiation system consisted of a semiconductor oscillator and applicator. (**B**) Block diagram of this system.

A novel microwave irradiation system was developed in cooperation with Sunny Engineering Co., Ltd (MTS03(S), Osaka, Japan) (Fig. 5). This system consists of a semiconductor microwave oscillator and an applicator. The semiconductor oscillator generates microwave outputs in the range of 0–20 W and a single and sharp frequency spectrum at 2.45 GHz (Fig. 5A). Figure 5B shows the block diagram for this system. Under a preset temperature inside the applicator (10, 20, or 30 °C was adopted in this study), microwave irradiation can be applied at different output powers to keep the temperature of cultured cells at 37 °C. In the applicator, temperatures and outputs were controlled as follows. During microwave irradiation, temperature of cultured cells was measured via infrared temperature sensors under the dishes, and the temperature inside the applicator was monitored using a thermistor. A Peltier module was used to control the temperature inside the applicator at 10–40 °C. The infrared temperature sensors and thermistors did not mutually interact, but an applicator fan was used to circulate the air once every 30 s. The thermal information from the infrared temperature sensors was transmitted to a controller once per second. Incident wave outputs were transmitted via a coaxial cable. The controller converted the data into VSWR (voltage standing wave ratio) and, based on the resulting data, it sent signals for the semiconductor oscillator to generate the next output value of the incident wave. The incident waves irradiation was provided by a patch antenna above the dishes. Information such as the outputs of the incident and reflected waves, VSWRs, and temperatures of the infrared temperature sensors and thermistor were recorded once per second and displayed monitor on the applicator. Potential microwave leakage was prevented by using an applicator made of aluminum. Additionally, The material of dish is Polystyrene, whose tanδ at 3.00 GHz is about 0.0003^32^. The tanδ of the dish is much lower than that of cells and media (Fig. 2B). Thus, the heat generation by microwave irradiation may efficiently occur in cells and media rather than in the dish.

**Figure 6:**
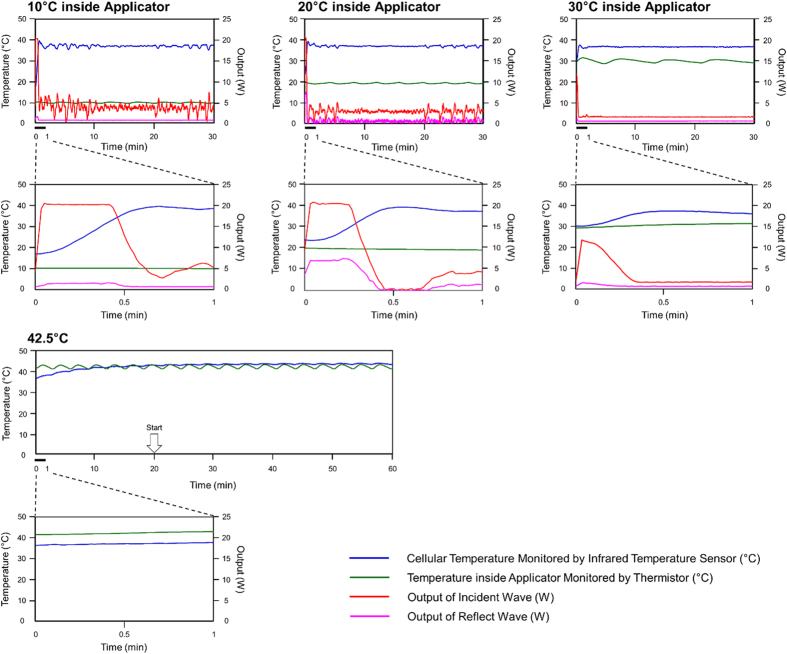
Changes in temperature and output of microwave irradiation. Microwave irradiation was applied for 0.5 h where the temperature inside the applicator was set at 10, 20, or 30 °C, while the temperature of the cultured cells in the dishes was set at 37 °C. The changes over 0–1 min are shown in the lower panels. Thermal treatment was applied for 1 h, while the temperature of the cultured cells in the dishes was set at 42.5 °C. The incubation time was started to count after dishes were incubated for 20 min, which the temperature was reached at 42.5 °C.

Microwaves were applied to cultured cells at a fixed temperature of 37 °C setting different temperatures in the applicator, i.e., 10, 20, and 30 °C (Fig. 6). The maximum output value of incident waves were set at 20 W. The initial transitions from 0–1 min for each applicator temperature were shown in the lower panels, and the mean values for the irradiation output (output value of the incident wave – output value of the reflected wave) except for the initial transition were 11.8 W (10 °C), 5.1 W (20 °C), and 2.7 W (30 °C). The temperature of the dishes measured by the infrared temperature sensor drifted over 37 °C from 0 to 1 min, and the values of the drifting temperatures were 39.6 °C (10 °C), 39.2 °C (20 °C), and 37.5 °C (30 °C). After initial irradiation, the irradiation output and temperatures reached a plateau. The drifting temperature of dishes under the incubation at 42.5 °C appears less than that induced by microwave irradiation. The mean values for the irradiation output were 3.1 W (10 °C), 2.3 W (20 °C), and 1.0 W (30 °C), and the mean temperature values were 37.0 °C (10 °C), 37.0 °C (20 °C), and 36.6 °C (30 °C) from 1 to 30 min.

Thus, the heating rate at initial irradiation time was faster as the temperature inside applicator became low. In order to verify the effects of the heating rate on cellular viability, we also investigated the viability of HL-60 cells, whereby the temperature inside the applicator was set at 10 °C and the maximum output value was set at 5 W (Fig. S1A). Under such conditions, the heating rate would be lower than any of the conditions shown in Fig. 6, suggesting that the effects of the heating rate would be smaller than that for any of those conditions. As shown in Fig. S1B, the viability of cells subjected to microwave irradiation for 1 h was decreased by a magnitude that was not less than that under microwave irradiation at 20 W. Thus, it is implied that the heating rate did not affect cellular viability, as observed in Fig. 6 of our study.

Moreover, we irradiated microwave to cells for 1 or 5 min under a setting of 10 °C inside the applicator with the maximum output value set at 20 W (Fig. S2). The viability of cells was not dramatically decreased and cells were killed in a time-dependent manner. Taking these additional results and observations into consideration, it is implied that the changes in temperature and output at initial irradiation time would not be the major cause of decreased cellular viability.

In order to determine whether the temperature of the media is almost the same as that of the dish, we measured the temperature in the suspension of HL-60 cells by a fiber-optic thermometer (Photon Control Inc., Burnaby, Canada) and an infrared temperature sensor (Fig. S3). The microwave irradiation condition was the same as that of the ‘10 °C inside applicator’ shown in Fig. 6. The temperature of each point in Fig. S3(A) was measured individually at four times. The mean temperature values from 0 to 30 min as measured by the fiber-optic thermometer were 37.3 °C, 32.9 °C, 39.0 °C, 36.6 °C, and 33.3 °C for points No. 1, No. 2, No. 3, No. 4, and No. 5, respectively. This suggests that the temperature distribution was different at each point. Therefore, we cannot confirm that the temperature did not exceed 42.5 °C anywhere in the cell suspension. However, the temperature at each point was not greater than 42.5 °C. If the temperature exceeded 42.5 °C anywhere in the cell suspension, the effects are most likely to be local and not have a major impact on the cellular viability.

### Viability of Cultured Cells under Microwave Irradiation

Each cell line was seeded in 35-mm dishes (Iwaki/Asahi Glass, Tokyo, Japan) in a volume of 2.5 mL with a density of 1 × 10^5^ cells/mL, HL-60; 5 × 10^4^ cells/mL, MCF-12A, MCF-7, MDA-MB-231, Panc-1, HGC-27, and KATO III; 1 × 10^4^ cells/mL, T98G. Adherent cells except for HL-60 and KATO III adhered to the dishes clearly. Microwave (MW) irradiation was applied for 1 h with the irradiation temperature maintained at 37 °C, while the temperature inside applicator was set at 10 °C. Following irradiation, adherent cells were lifted using 0.5% trypsin-EDTA (Thermo Fisher Scientific, Yokohama, Japan), and cells were plated in 96-well flat-bottom plastic plates (100 μL per well) and incubated for 24, 48, or 72 h in a 5% CO_2_ incubator at 37 °C before further viability assays were performed. Negative control cells were incubated at 37 °C in a CO_2_ incubator instead of microwave irradiation, and then incubated in a CO_2_ incubator for 24, 48, or 72 h. The cells of ‘42.5 °C’ were incubated at 42.5 °C in applicator as a thermal treatment, and then incubated in CO_2_ incubator for 24, 48, or 72 h. Viability was assessed using the water-soluble tetrazolium salt WST-8 (Cell Count Reagent SF; Nacalai Tesque, Kyoto, Japan) according to the manufacturer’s instructions. WST-8 reagent (10 μL) was added to the medium of cells that had been plated in 96-well plates after microwave irradiation and incubated for 2 h at 37 °C following the total incubation time. The amount of formazan product generated by the WST-8 assay was quantified by spectrophotometric measurement at 450 nm using a microplate reader (iMark; Bio-Rad Laboratories, Hercules, USA).

### Dielectric Properties of Cultured Cells

The relative permittivity and dielectric loss of cell suspensions, cell clusters, and medium for each cell line, PBS and ultra-pure water at 37 °C were measured by the coaxial probe method using an a Agilent Technologies N5242A Network Analyzer with a Dielectric Probe Kit, 85070E, in a range of 500 MHz to 20 GHz (Agilent Technologies Inc., Santa Clara, USA). The concentration of each cell suspension was 1 × 10^5^ cells/mL for HL-60 cells; 5 × 10^4^ cells/mL for MCF-12A, MCF-7, MDA-MB-231, Panc-1, HGC-27, and KATO III cells; and 1 × 10^4^ cells/mL for T98G cells. Cell clusters were prepared wherein cells were centrifuged at 1000 rpm for 5 min, and then washed in PBS twice. The dissipation factor, tanδ, was calculated using the equation.

tanδ = ε’/ε”, where ε’ and ε” are the relative permittivity and dielectric loss, respectively. All values reflect the mean of four determinations, where five measurements were obtained for each determination.

### Viability of HL-60 cells Under Various Microwave Irradiation Conditions

HL-60 cells were seeded in 35-mm dishes in a volume of 2.5 mL with a density of 1 × 10^5^ cells/mL. MW irradiation was applied for 0, 0.5, 1, 2 and 3 h with the irradiation temperature maintained at 37 °C and the temperature inside the applicator set to 10, 20 or 30 °C. After irradiation, cells were incubated in a CO_2_ incubator for 24, 48, and 72 h, and viability was assessed as described above.

### Effects of heating rate under microwave irradiation on viability of HL-60 cells

HL-60 cells were seeded in 35-mm dishes in a volume of 2.5 mL with a density of 1 × 10^5^ cells/mL. MW irradiation was applied for 1 h with the irradiation temperature maintained at 37 °C and the temperature inside the applicator set to 10 °C. The maximum output value of incident waves were set at 5 W. After irradiation, cells were incubated in a CO_2_ incubator for 24, 48, and 72 h, and viability was assessed as described above.

### Effects of microwave irradiation at initial time on viability of HL-60 cells

HL-60 cells were seeded in 35-mm dishes in a volume of 2.5 mL with a density of 1 × 10^5^ cells/mL. MW irradiation was applied for 1 or 5 min with the irradiation temperature maintained at 37 °C and the temperature inside the applicator set to 10 °C. The maximum output value of incident waves were set at 20 W. After irradiation, cells were incubated in a CO_2_ incubator for 24, 48, and 72 h, and viability was assessed as described above.

### Statistical Analysis

All values in Fig. 1,3, S1 and S2 reflect the mean of four determinations ± SD, and one-way analysis of variance (one-way ANOVA) was performed, followed by Tukey test analysis. The comparisons of the analysis were among the negative control, MW treatment, and 42.5 °C thermal treatment in Fig. 1, among irradiation times or output values in Fig. 3, S1 or S2. Figure 4B shows the linear regression curve and its equation. In all cases, *P* < 0.05 was considered statistically significant.

## Additional Information

**How to cite this article**: Asano, M. *et al*. Effects of Normothermic Conditioned Microwave Irradiation on Cultured Cells Using an Irradiation System with Semiconductor Oscillator and Thermo-regulatory Applicator. *Sci. Rep.* **7**, 41244; doi: 10.1038/srep41244 (2017).

**Publisher's note:** Springer Nature remains neutral with regard to jurisdictional claims in published maps and institutional affiliations.

## Supplementary Material

Supplementary Information

## References

[b1] Mai H.-Q. . Endoscopic microwave coagulation therapy for early recurrent T1 nasopharyngeal carcinoma. European Journal of Cancer 45, 1107–1110 (2009). 19327985 10.1016/j.ejca.2009.02.028

[b2] Tabuse K. . Microwave surgery: hepatectomy using a microwave tissue coagulator. World journal of surgery 9, 136–142 (1985). 3984365 10.1007/BF01656265

[b3] Hildebrandt B. . The cellular and molecular basis of hyperthermia. Critical reviews in oncology/hematology 43, 33–56 (2002). 12098606 10.1016/s1040-8428(01)00179-2

[b4] Hornback N. Historical aspects of hyperthermia in cancer therapy. Radiologic Clinics of North America 27, 481–488 (1989). 2648453

[b5] Wust P. . Hyperthermia in combined treatment of cancer. The lancet oncology 3, 487–497 (2002). 12147435 10.1016/s1470-2045(02)00818-5

[b6] Herrero M. A., Kremsner J. M. & Kappe C. O. Nonthermal microwave effects revisited: on the importance of internal temperature monitoring and agitation in microwave chemistry. The Journal of organic chemistry 73, 36–47 (2008). 18062704 10.1021/jo7022697

[b7] Horikoshi S., Fukui M., Tsuchiya K., Abe M. & Serpone N. Microwave specific effects in organic synthesis: A proposed model from the solvent-free synthesis of monoglycerylcetyldimethylammonium chloride. Chemical Physics Letters 491, 244–247 (2010).

[b8] Kappe C. O. Controlled microwave heating in modern organic synthesis. Angewandte Chemie International Edition 43, 6250–6284 (2004). 15558676 10.1002/anie.200400655

[b9] Ballardin M. . Non-thermal effects of 2.45 GHz microwaves on spindle assembly, mitotic cells and viability of Chinese hamster V-79 cells. Mutation Research/Fundamental and Molecular Mechanisms of Mutagenesis 716, 1–9 (2011). 21827772 10.1016/j.mrfmmm.2011.07.009

[b10] Cleary S. F., Cao G. & Liu L.-M. Effects of isothermal 2.45 GHz microwave radiation on the mammalian cell cycle: comparison with effects of isothermal 27 MHz radiofrequency radiation exposure. Bioelectrochemistry and bioenergetics 39, 167–173 (1996).

[b11] Lantow M., Viergutz T., Weiss D. & Simko M. Comparative study of cell cycle kinetics and induction of apoptosis or necrosis after exposure of human mono mac 6 cells to radiofrequency radiation. Radiation research 166, 539–543 (2006). 16953672 10.1667/RR3601.1

[b12] Lee S. . 2.45 GHz radiofrequency fields alter gene expression in cultured human cells. Febs letters 579, 4829–4836 (2005). 16107253 10.1016/j.febslet.2005.07.063

[b13] Leszczynski D., Joenväärä S., Reivinen J. & Kuokka R. Non‐thermal activation of the hsp27/p38MAPK stress pathway by mobile phone radiation in human endothelial cells: Molecular mechanism for cancer‐and blood‐brain barrier‐related effects. Differentiation 70, 120–129 (2002). 12076339 10.1046/j.1432-0436.2002.700207.x

[b14] Lim H. B., Cook G. G., Barker A. T. & Coulton L. A. Effect of 900 MHz electromagnetic fields on nonthermal induction of heat-shock proteins in human leukocytes. Radiation research 163, 45–52 (2005). 15606306 10.1667/rr3264

[b15] Lixia S. . Effects of 1.8 GHz radiofrequency field on DNA damage and expression of heat shock protein 70 in human lens epithelial cells. Mutation Research/Fundamental and Molecular Mechanisms of Mutagenesis 602, 135–142 (2006). 17011595 10.1016/j.mrfmmm.2006.08.010

[b16] Merola P. . Proliferation and apoptosis in a neuroblastoma cell line exposed to 900 MHz modulated radiofrequency field. Bioelectromagnetics 27, 164–171 (2006). 16437547 10.1002/bem.20201

[b17] Peinnequin A. . Non-thermal effects of continuous 2.45 GHz microwaves on Fas-induced apoptosis in human Jurkat T-cell line. Bioelectrochemistry 51, 157–161 (2000). 10910164 10.1016/s0302-4598(00)00064-7

[b18] Yu Y. & Yao K. Non-thermal cellular effects of low-power microwave radiation on the lens and lens epithelial cells. Journal of International Medical Research 38, 729–736 (2010). 20819410 10.1177/147323001003800301

[b19] Zhao R. . Studying gene expression profile of rat neuron exposed to 1800MHz radiofrequency electromagnetic fields with cDNA microassay. Toxicology 235, 167–175 (2007). 17449163 10.1016/j.tox.2007.03.015

[b20] Szasz O. Essentials of Oncothermia. Conference Papers in Science 2013, 1–20 (2013).

[b21] Andocs G., Szasz O. & Szasz A. Oncothermia treatment of cancer: from the laboratory to clinic. Electromagnetic Biology and Medicine 28, 148–165 (2009). 19811397 10.1080/15368370902724633

[b22] Szasz A. Hyperthermia, a modality in the wings. Journal of Cancer Research and Therapeutics 3, 56 (2007). 17998724 10.4103/0973-1482.31976

[b23] Mori I. . Microwave cell death: Molecular analysis using DNA electrophoresis, PCR amplification and TUNEL. Pathology international 59, 294–299 (2009). 19432670 10.1111/j.1440-1827.2009.02368.x

[b24] Ozaki T. . Microwave cell death: Enzyme histochemical evaluation for metastatic carcinoma of the liver. Pathology international 53, 837–845 (2003). 14629749 10.1046/j.1440-1827.2003.01571.x

[b25] Ogurof T. Trends in Magnetrons for Consumer licrowave Owens. Journal of Microwave Power 13, 1 (1978).

[b26] Osepchuk J. M. Microwave power applications. Microwave Theory and Techniques, IEEE Transactions on 50, 975–985 (2002).

[b27] Abe M. . Multi‐institutional studies on hyperthermia using an 8‐MHz radiofrequency capacitive heating device (thermotron RF‐8) in combination with radiation for cancer therapy. Cancer 58, 1589–1595 (1986). 3756783 10.1002/1097-0142(19861015)58:8<1589::aid-cncr2820580802>3.0.co;2-b

[b28] Raoof M. . Tumor selective hyperthermia induced by short-wave capacitively-coupled RF electric-fields. PloS one 8, e68506 (2013). 23861912 10.1371/journal.pone.0068506 PMC3701653

[b29] Bonner W. M. Low-dose radiation: thresholds, bystander effects, and adaptive responses. Proceedings of the National Academy of Sciences 100, 4973–4975 (2003). 10.1073/pnas.1031538100 PMC154280 12704228

[b30] Chinnaiyan A. M. . Combined effect of tumor necrosis factor-related apoptosis-inducing ligand and ionizing radiation in breast cancer therapy. Proceedings of the National Academy of Sciences 97, 1754–1759 (2000). 10.1073/pnas.030545097 PMC26508 10677530

[b31] Dewey W. C., Ling C. C. & Meyn R. E. Radiation-induced apoptosis: relevance to radiotherapy. International Journal of Radiation Oncology* Biology* Physics 33, 781–796 (1995). 10.1016/0360-3016(95)00214-8 7591884

[b32] Hippel A. v. Dielectric materials and applications. London: Artech House (1954).

