# Supplementary material for: Effects of Normothermic Conditioned Microwave Irradiation on Cultured Cells Using an Irradiation System with Semiconductor Oscillator and Thermo-regulatory Applicator
Source: Sci Rep. 2017 Feb 1;7:41244. doi: 10.1038/srep41244 (PMC5286535; doi:10.1038/srep41244)
Supplement: Supplementary Information [file srep41244-s1.pdf]

Effects of Normothermic Conditioned Microwave Irradiation on Cultured Cells Using an Irradiation System with Semiconductor Oscillator and Thermo-regulatory Applicator

Mamiko Asano[1]*, Minoru Sakaguchi[1], Satoshi Tanaka[1], Keiichiro Kashimura[2], Tomohiko Mitani[3], Masaya Kawase[4], Hitoshi Matsumura[1], Takako Yamaguchi[1], Yoshikazu Fujita[1], and Katsuyoshi Tabuse[1]

[1]Faculty of Pharmaceutical Sciences, Osaka University of Pharmaceutical Sciences, 4-20-1 Nasahara, Takatsuki, Osaka 569-1094, Japan

[2]Faculty of Engineering, Chubu University, 1200 Matsumoto-cho, Kasugai, Aichi 487-8501, Japan

[3]Research Institute for Sustainable Humanosphere, Kyoto University, Gokasho, Uji, Kyoto, 611-0011, Japan

[4]Department of Bioscience, Nagahama Institute of Bio-Science and Technology, 1266 Tamura-cho, Nagahama, Shiga 526-0829, Japan

* Correspondence author

Email: asanom@gly.oups.ac.jp

Supplementary Figure Legends

Figure S1. Effects of heating rate under microwave irradiation on viability of HL-60 cells. (A) Changes in temperature and output of microwave irradiation at the maximum output value of incident waves at 5 W. Microwave irradiation was applied for 0.5 h where the temperature inside the applicator was set at 10°C, while the temperature of the cultured cells in the dishes was set at 37°C. The changes over 0–1 min are shown in the lower panels. (B) Microwave irradiation (described as 'MW') was applied for 1 h with the temperature of the cultured cells maintained at 37°C, whereas the temperature inside the applicator was set at 10°C. During irradiation, output could be generated at the maximum value of 20 and 5 W. After the irradiation ceased, cells were moved to a CO_2 incubator and incubated for 24, 48, or 72 h. The negative control cells were incubated at 37°C in a CO_2 incubator rather than being subjected to microwave irradiation. Cellular viability was determined using the WST-8 assay. Rates are shown relative to the absorbance of the negative control, whose values were defined as "100". The horizontal axes indicate the duration of incubation after irradiation. Data are expressed as the mean ± SD of four independent experiments. Asterisks indicate significant differences from the negative control: $^{*}P < 0.05$, $^{**}P < 0.01$. The results of 'MW (20 W)' are the same as that of 'MW (10°C inside Applicator)' for irradiation time of 1 h in Fig. 3.

Figure S2. Effects of microwave irradiation at initial irradiation time on viability of HL-60 cells. Microwave irradiation (described as 'MW') was applied for 1 or 5 min with the temperature of the cultured cells maintained at 37°C, whereas the temperature inside the applicator was set at 10°C. During irradiation, output could be generated at the maximum value of 20 W. After the irradiation ceased, cells were moved to a CO_2 incubator and incubated for 24, 48, or 72 h. The negative control cells were incubated at 37°C in a CO_2 incubator rather than being subjected to microwave irradiation. Cellular viability was determined using the WST-8 assay. Rates are shown relative to the absorbance of the negative control, whose values were defined as "100". The horizontal axes indicate the duration of incubation after irradiation. Data are expressed as the mean ± SD of four independent experiments. Asterisks indicate significant differences from the negative control: $^{*}P < 0.05$, $^{**}P < 0.01$. The results

of ‘MW (1 h)’ are the same as that of ‘MW (10°C inside Applicator)’ for irradiation time of 1 h in Fig. 3.

Figure S3. The Temperature of HL-60 cells Measured by a Fiber-optic Thermometer.
(A) The five points which were measured temperatures by a fiber-optic thermometer. (B) Changes in temperatures for 0.5 h where the temperature inside the applicator was set at 10°C, while the temperature of the cultured cells in the dishes was set at 37°C. The changes over 0–1 min are shown in the lower panels. The condition of microwave irradiation was the same as that for ‘10°C inside applicator’ in Fig. 6, and the temperature of each point was measured individually at four times.

Figure S1.

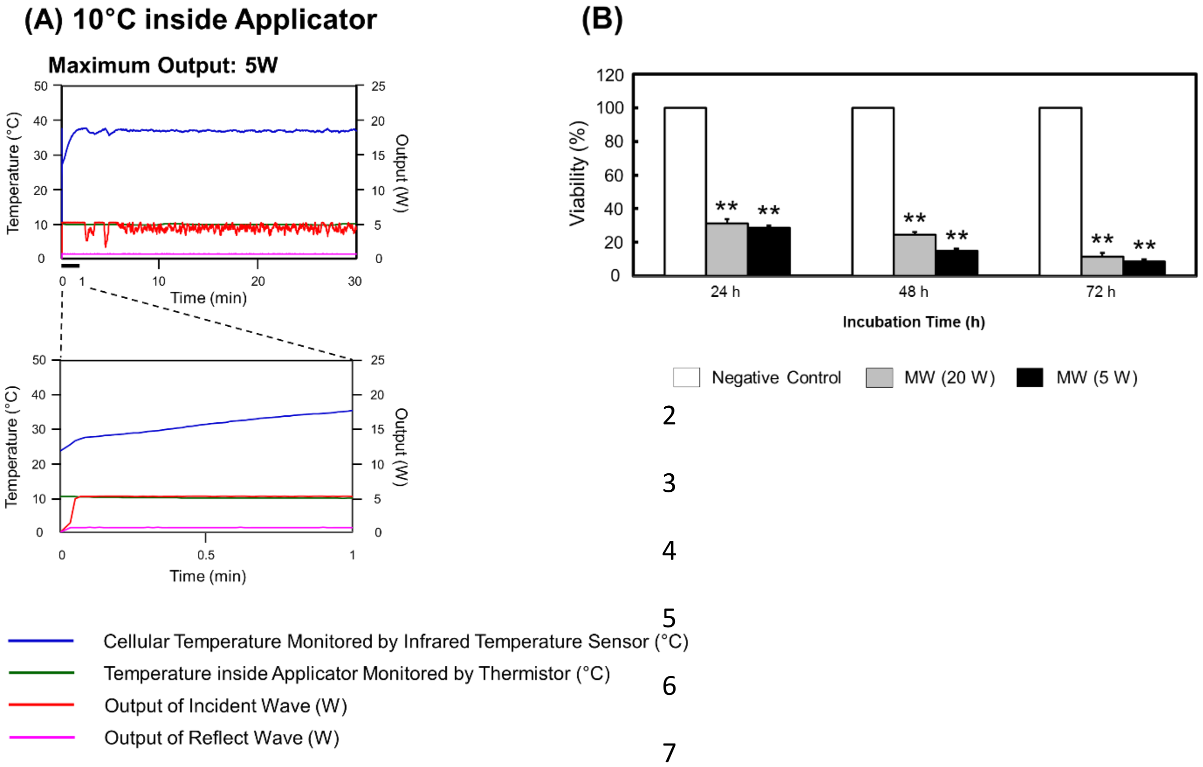
(A) 10°C inside Applicator
Maximum Output: 5W
Temperature (°C)
Output (W)
Time (min)
(B)
Viability (%)
24 h
48 h
72 h
Incubation Time (h)
Negative Control
MW (20 W)
MW (5 W)
Cellular Temperature Monitored by Infrared Temperature Sensor (°C)
Temperature inside Applicator Monitored by Thermistor (°C)
Output of Incident Wave (W)
Output of Reflect Wave (W)

Figure S2.

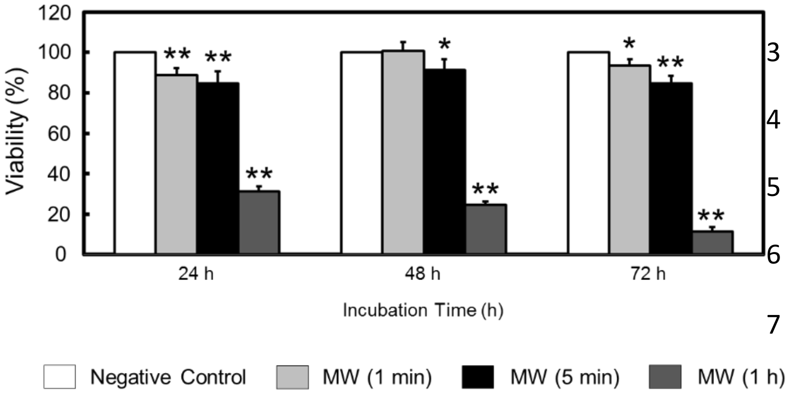

Viability (%)
120
100
80
60
40
20
0
**
**
**
*
**
*
**
**
24 h
48 h
72 h
Incubation Time (h)
Negative Control
MW (1 min)
MW (5 min)
MW (1 h)

Figure S3.

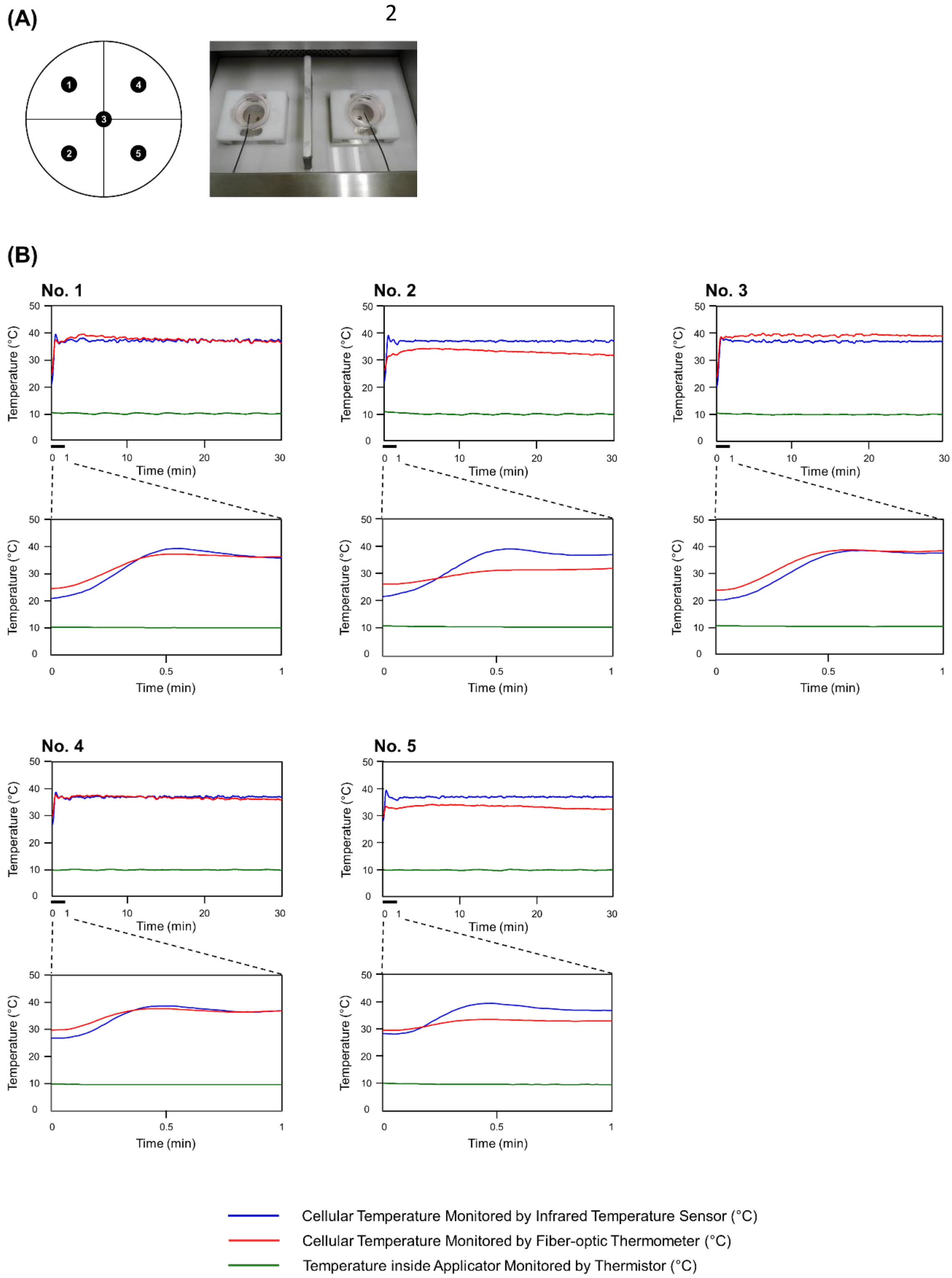
(A)
1
2
3
4
5
(B)
No. 1
No. 2
No. 3
No. 4
No. 5
Temperature (°C)
Time (min)
Cellular Temperature Monitored by Infrared Temperature Sensor (°C)
Cellular Temperature Monitored by Fiber-optic Thermometer (°C)
Temperature inside Applicator Monitored by Thermistor (°C)
